# Gene Silencing of 4-1BB by RNA Interference Inhibits Acute Rejection in Rats with Liver Transplantation

**DOI:** 10.1155/2013/192738

**Published:** 2013-01-31

**Authors:** Yang Shi, Shuqun Hu, Qingwei Song, Shengcai Yu, Xiaojun Zhou, Jun Yin, Lei Qin, Haixin Qian

**Affiliations:** ^1^Department of General Surgery, The Affiliated Hospital of Xuzhou Medical College, Xuzhou, Jiangsu 221002, China; ^2^Department of General Surgery, The First Affiliated Hospital of Soochow University, Suzhou, Jiangsu 215006, China

## Abstract

The 4-1BB signal pathway plays a key role in organ transplantation tolerance. In this study, we have investigated the effect of gene silencing of 4-1BB by RNA interference (RNAi) on the acute rejection in rats with liver transplantation. The recombination vector of lentivirus that contains shRNA targeting the 4-1BB gene (LV-sh4-1BB) was constructed. The liver transplantation was performed using the two-cuff technique. Brown-Norway (BN) recipient rats were infected by the recombinant LVs. The results showed that gene silencing of 4-1BB by RNAi downregulated the 4-1BB gene expression of the splenic lymphocytes *in vitro*, and the splenic lymphocytes isolated from the rats with liver transplantation. LV-sh4-1BB decreased the plasma levels of liver injury markers including AST, ALT, and BIL and also decreased the level of plasma IL-2 and IFN-**γ** in recipient rats with liver transplantation. Lentivirus-mediated delivery of shRNA targeting 4-1BB gene prolonged the survival time of recipient and alleviated the injury of liver morphology in recipient rats with liver transplantation. In conclusion, our results demonstrate that gene silencing of 4-1BB by RNA interference inhibits the acute rejection in rats with liver transplantation.

## 1. Introduction

Costimulatory pathways between antigen-presenting cells (APCs) and Tcells play a crucial role in T-cells activation and alloimmune responses [1, 2]. 4-1BB is a member of the tumor necrosis factor receptor superfamily. The functions of receptor mainly as a costimulatory molecule in T lymphocytes. 4-1BB is expressed in activated T cells and antigen presenting cells such as dendritic cells [3]. 4-1BB/4-1BB ligand (4-1BBL) signaling provides T-cells activation with costimulation, that is, dependent or independent of CD28 [4]. 4-1BB can supply sufficient costimulatory signals for T-cells activation [5]. Under the condition of repeated antigen stimulation, the downregulationg expresion of CD28 protein in activated T cells results in activation-induced celll death (AICD), whereas very few 4-1BB molecules may supply sufficient costimulatory signals to sustain T-cells activation and inhibit AICD [6]. 

As we all know, the liver transplantation is an effective therapy for end-stage liver disease. But the transplantation rejective response is a formidable problem after liver transplantation, especially acute rejection which is the main cause of early dysfunction and transplantation failure [7]. The incidence rate of acute rejection after liver transplantation is up to 50% to 70% [8]. The acute rejection after the organ transplantation is mainly caused by the activation of the immune system in the host. During the acute rejection, T cells are the most important effectors involved in antigen recognition, lymphcyte proliferation and differentiation, and immune regulation, cytolysis. 4-1BB plays a critical role in allograft rejection [9, 10]. Our previous study illuminated that the blockade of the 4-1BB/4-1BBL costimulatory pathway with 4-1BBL monoclonal antibody decreased the acute rejection in rat with liver transplantation [11].

RNA interference- (RNAi-) mediated gene silencing can be generated by the expression of a vector-mediated RNAi anywhere in the genome and is already being tested as a potential therapy in clinical trials for a number of diseases [12, 13]. The viral-based vectors have been developed as an alternative strategy of gene therapy. Therefore, the aim of present study was to investigate the effect of gene silencing of 4-1BB by RNA interference on the acute rejection in rats with liver transplantation.

## 2. Materials and Methods

### 2.1. Lentiviral Vectors

The design of siRNA was performed according to the previously published guidelines by using the Ambion siRNA-finding software [14]. To minimize off-target effects, a BLAST homology search was systematically performed to ensure that a single-mRNA sequence was targeted. Replication-deficient, self-inactivating lentiviral vectors pcDNA-CMV-sh4-1BB-Lentivector (LV-sh4-1BB) and the empty vector (LV-NC) were generated as follows. As shown in Table 1, the target sequence of siRNA sequences targeting 4-1BB of rat (GenBank: NM-001025773) and control were as follows: shRNA1 (11–31), GCTGTTACAACATGGTGGTCA; shRNA2 (110–130), GCAGTAAATACCCTCCGGTCT; shRNA3 (191–211), GCAGAGTGTGTCAAGGCTATT; nonsilencing siRNA, ATTGGACCAAGTGGTTCATAGC. The oligonucleotides were designed according to the structure of the siRNA sense strand-loop-siRNA antisense strand. The siRNA sequences targeting 4-1BB of rat were shown in Table 1. The oligonucleotides were synthesized and used for the construction of pcDNA-CMV-sh4-1BB-Lentivector. A negative control (LV-NC) with DNA oligos targeting 4-1BB of rat was also designed.

The shRNAs were cloned into lentiviral work vector pcDNA-CMV-Lentivector (Shanghai GeneChem Co. Ltd., Shanghai, China), which was linearized using restriction endonucleases BamH I and Pst I. All constructs were verified by sequence analysis. The recombinant lentiviral vectors were designated as LV-sh4-1BB1, LV-shP4-1BB2, LV-shP4-1BB3, and LV-NC. The recombinant work vector and package plasmids were cotransduced into 293T cells using Lipofectamine 2000 (Invitrogen, Carlsbad, CA, USA) for generating the lentivirus. After the cells were cultivated for 48 h, the culture medium was collected, concentrated by ultracentrifugation, aliquoted, and stored at −80°C until used. Virus titer is the number of cells expressing green fluorescent protein (GFP) multiplied by the corresponding dilution. The titer of lentivirus was determined by hole-by-dilution titer assay. The final titer of recombinant virus was 2 × 10^9^ transducing units (TU)/mL.

### 2.2. 293T-Cell Culture and Transduction

The 293T packaging cell line (Academy of Life Science, Shanghai, China) was cultivated in Dulbecco's modified Eagle's medium (DMEM) (Invitrogen, Carlsbad, CA, USA) supplemented with 10% fetal bovine serum (FBS) (Invitrogen, Carlsbad, CA, USA), 100 mg/mL streptomycin and 100 U/mL penicillin at 37°C in a humidified incubator containing 5% CO_2_. Cells were seeded in 24-well plates at 50–70% confluence 24 h prior to transduction. To analyze the transduction efficiency, 293T cells were gated to determine the percentage of GFP-positive cells. Cells with >85% viability were cultivated for additional experiments. 

### 2.3. T-Lymphocytes Isolation, Culture, and Transduction

Spleens were harvested from Brown-Norway (BN) rats (Animal Center of Soochow University, Suzhou, China) euthanazed by cervical dislocation. The procedures were performed according to the local guidelines for animal research approved by the Administrative Committee of Experimental Animal Care and Use of Soochow University. Single-cell suspensions were prepared by forcing tissue through a fine wire mesh using a syringe plunger followed by repeated pipetting in culture medium. RBC depletion involved cell lysis in 5 mL lysing buffer [0.14 moL/L NH_4_Cl, 0.017 moL/L Tris-base (pH 7.5)] for 5 min at 20°C followed by three washes in ice-cold medium. To activated T cell, phytohemagglutinin (50 *μ*g/mL) was added in the culture medium [15].

The T lymphocytes from BN rats were cultivated in RPMI 1640 culture medium (Invitrogen, Carlsbad, CA, USA) supplemented with 10% fetal calf serum (FCS) (Invitrogen, Carlsbad, CA, USA). Transient transduction was performed using Lipofectamine 2000 (Invitrogen, Carlsbad, CA, USA) according to the manufacturer's instructions in six- or 12-well plates with cells at 70–90% confluence.

### 2.4. Real-Time Quantitative PCR Analysis

Total RNA was extracted by using Trizol reagent (Invitrogen, Carlsbad, CA, USA) according to the manufacturer's instructions. Briefly, the splenic lymphocyte was treated with 1.5 mL Trizol reagent and kept at room temperature for 5 min. After the cells were incubated with 600 *μ*L chloroform for 3 min, the total RNA was centrifuged for 20 min at 12,000 g and 4°C. The total RNA was precipitated with 1000 *μ*L isopropanol. After incubation of 10 min at room temperature, the RNA was precipitated for 10 min at 16.000 g and 4°C. The resulting RNA-pellet was washed with 2000 *μ*L isopropanol. The concentration of RNA was assessed photometrically at 260 nm. The reactions were run on a Roche light Cycler Run 5.32 Real-Time PCR System (Bio-Rad, Hercules, CA, USA) with the following cycle conditions: 95°C for 15 sec, 45 cycles at 95°C for 10 sec, and at 60°C for 30 sec. Melt curve analyses of all real-time PCR products were performed and shown to produce a single-DNA duplex. Quantitative measurements were determined using the ΔΔCt method and expression of *β*-actin was used as the internal control. The mRNA expression of the control group was expressed as 100%. Fold-induction of mRNA expression was calculated [16]. The sequences of the 4-1BB gene primers were as follows: forward primer, 5'-GTGTCAAGGCTATTTCAG-3'; reverse primer, 5'-AGACCACGTCTTTCTCC-3'. the product was 275 bp. *β*-Actin served as a control for normalization. The primers were as follows: forward primer, 5'-CCTCATGAAGATCCTGACCG-3'; reverse primer, 5'-AGCCAGGGCAGTAATCTCCT-3'. The product was 488 bp.

### 2.5. Western Blot Analysis

The splenic lymphocytes were collected. Cells were washed with cold phosphate-buffered saline (PBS) containing 2 mmoL/L EDTA and lysed with denaturing SDS-PAGE sample buffer using standard methods. Protein extract was prepared. The protein concentration in supernatant was determined by the BCA assay. The final concentration of protein in each sample was adjusted to 2 mg/mL. Protein lysates were separated by 12% SDS-PAGE and transferred onto polyvinylidene fluoride membrane (Millipore, Bedford, MA). The membranes were blocked with 5% skim milk for 1 h at 37°C. Then the membranes were incubated with rabbit anti-4-1BB monoclonal antibody (dilution at 1 : 200) and anti-*β*-actin antibody (dilution at 1 : 300) (Santa Cruz Biotechnology, Santa Cruz, CA, USA) at 48°C overnight. After the membranes were washed three times with Tris-buffered saline, they were incubated with horseradish peroxidase (HRP)—conjugated goat anti-rabbit immunoglobulin G (IgG) antibody (dilution at 1 : 1,000) (Santa Cruz) at room temperature for 4 h. Immunoreactivity was detected by enhanced chemiluminescence (ECL). The band density was measured with Quantity One analysis software (Bio-Rad, Hercules, CA, USA), and the quantification of 4-1BB to *β*-actin levels was done by densitometry analysis [17].

### 2.6. Animals and Rat Liver Transplantation Models

Inbred male Lewis (LEW) and BN rats weighing 200–250 g were purchased from the Vital River Laboratories (Beijing, China). All animals were housed under conditions of constant temperature (22°C) and humidity in a specific-pathogen-free facility. The rats were fed with the commercial rat chow pellets. Male LEW rats were used as the liver donors and BN rats as the recipients.

Orthotopic liver transplantation was performed using the “two-cuff technique” as previously described [11, 18]. Briefly, the donors and recipients were anesthetized with the ether. The suprahepatic vena cava was reconstructed using continuous 8-0 polypropylene sutures. The hepatic artery was not reconstructed. The portal vein was reanastomosed using a polyethylene cuff (8F). When the anastomosis of the portal vein and suprahepatic vena cava was completed, the liver was reperfused. The anastomosis of the infrahepatic vena cava was then completed by the same cuff technique (6F). The bile duct was anastomosed with an intraluminal polyethylene stent (22G). The transplantation procedure required less than 60 min, during which the portal vein was clamped for 13 to 15 min. The procedures were performed according to the local guidelines for animal research approved by the Administrative Committee of Experimental Animal Care and Use of Soochow University.

### 2.7. Experimental Animal Grouping

Sixty four BN rats were randomly divided into four groups: sham group (S), liver transplantation model group (M), LV-sh4-1BB1 group (LV-sh4-1BB1), and LV-NC group (LV-NC). The rats of the sham and model group were injected 1 mL saline via the dorsal penis vein, respectively, before operation. The rats of the LV-sh4-1BB1 group and LV-NC group were injected 1 mL recombinant LVs and empty LVs via the dorsal penis vein, respectively, before liver transplantation.

On the 7th day after transplantation, 8 rats in each group were killed by cervical dislocation. The lymphocytes were obtained from spleen and used for measurement of 4-1BB expression by real-time RT-PCR and Western blot analysis. Blood samples were gathered by the inferior vena cava and used for biochemistry tests and cytokine assay. The liver lobes were excised to study the pathological changes. The remanent rats in each group were used to recored the survival time of posttransplantation.

### 2.8. Analysis of Plasma Liver Function Markers and Cytokines

On the 7th day after transplantation, serum aspartate transaminase (AST), alanine aminotransferase (ALT), lactate dehydrogenase (LDH), and bilirubin (BIL) were measured by using the automatic biochemical meter (TMS1024, Tokyo Bokei, Japan). The concentrations of interleukin-2 (IL-2), IL-10, and interferon-*γ* (IFN-*γ*) in plasma were tested using enzyme-linked immunosorbent assay (ELISA) kits (Biosource International, Inc. Camarillo, CA, USA). All the procedures were performed according to the instruction of the manufacturers.

### 2.9. Histological and Morphometric Analysis of Liver Grafts

On the 7th day posttransplantation, recipient rats were sacrificed. The grafted liver samples were fixed in 10% formalin and embedded in paraffin. Five micrometer thick sections were affixed to slides, deparaffinized, and stained with hematoxylin and eosin. The severity of acute rejection was assessed in a blinded fashion with a rejection activity index (RAI) according to Banff criterion [11, 19, 20].

Grafted livers were fixed in 4% glutaraldehyde, dehydrated with ethanol, and then embedded in Epon812. Liver sections were sliced and stained with uranium lead. Microstructure was read using Hitachi H-600 electron microscopy (Hitachi, Japan).

### 2.10. Statistical Analysis

Data are presented as mean ± standard deviation (SD). The statistical evaluation of differences in recipient survival was performed using the log rank test applied to Kaplan-Meier plots. All other statistical comparisons among groups were conducted using analysis of variance (ANOVA) with subsequent Dunnett's *t-*test. Significance was defined as *P* < 0.05.

## 3. Results

### 3.1. PCR Identification of Constructed shRNA Expression Plasmids and Titer Determination and Packaging of the Lentiviral Vector

**Figure 1:**
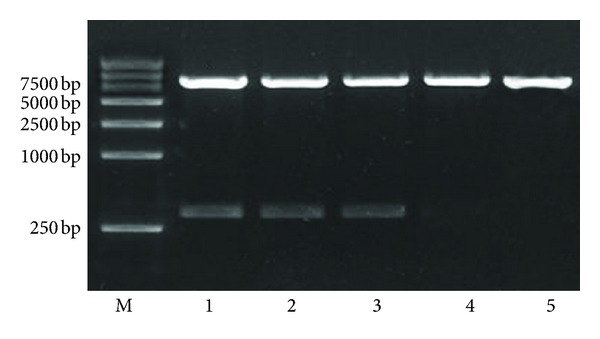
The identification of constructed shRNA expression plasmids by restriction enzyme. Lane 1: LV-sh4-1BB1; lane 2: LV-sh4-1BB2; lanes 3: LV-sh4-1BB3; lanes 4: empty pcDNA-CMV-Lentivector (blank-control); 5: negative-control shRNA (LV-NC).

The pcDNA-CMV-Lentivector 4-1BB shRNA expression plasmids were identified using a restriction endonuclease digestion. The lengths of pcDNA-CMV-Lentivector is 7.8 kb and pcDNA-CMV-Lentivector contains BamH I and Pst I two restriction sites. The product of pcDNA-CMV-Lentivector is linear large fragment after the double enzyme. The product of the recombination vector of lentivirus containing shRNA targeting the 4-1BB gene were two fragments, one was 7.8 kb and the other was 326 bp. As shown in Figure 1, results of DNA sequencing were as expected. The recombination vector of lentivirus encoding the specially designed shRNA against the 4-1BB gene was named pcDNA-CMV-sh4-1BB-Lentivector (LV-sh4-1BB). The empty vector was used as blank-control and named pcDNA-CMV-Lentivector (LV-BC). The negative-control shRNA was named LV-NC.

Forty-eight hours after cotransfection of the three-plasmid lentiviral vector into 293T cells, strong green fluorescence was observed using an inverted fluorescence microscope. After a single exposure of 293T cells to the lentivirus, a high percentage (>90%) of transfectants expressed GFP at 48 h after the transduction, indicating a high and stable transduction of the lentiviral vector system (data not shown).

### 3.2. Efficiency of Lentiviral Transfection into the Splenic Lymphocytes

**Figure 2:**
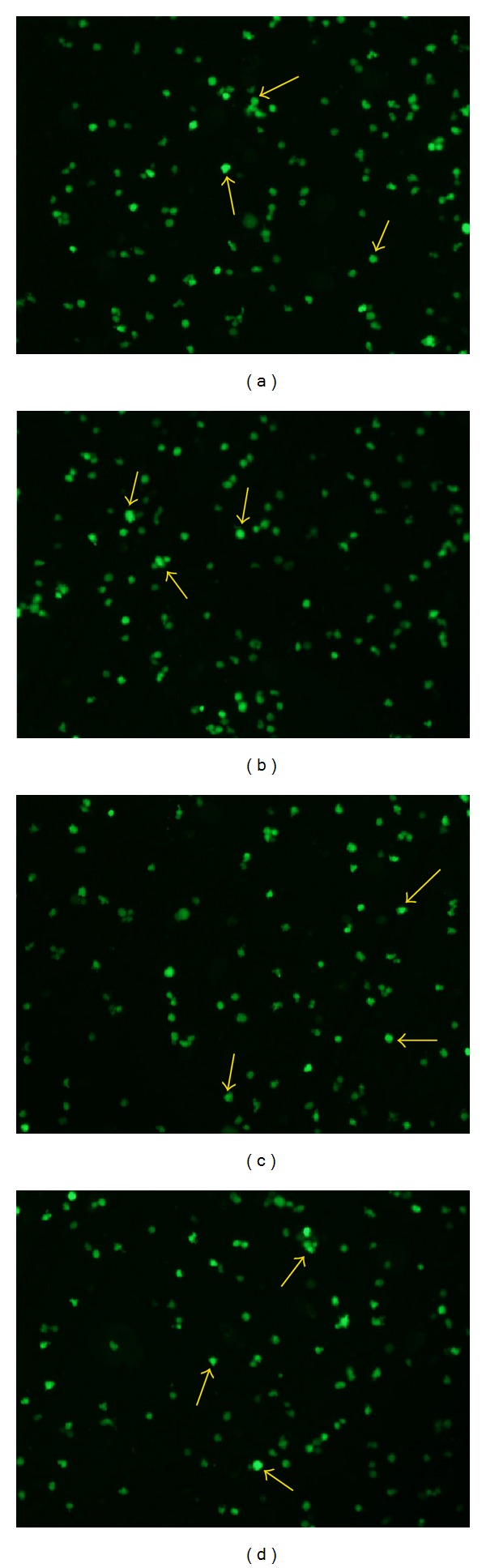
Representative fluorescence microscopy images of splenic lymphocytes 72 h after transduction with pcDNA-CMV-sh4-1BB-Lentivector1-3 (LV-sh4-1BB1-3) (a–c) and negative-control shRNA group (LV-NC) (d) (×100). Arrows indicate GFP fluorescence in the splenic lymphocytes.

To demonstrate the efficiency of siRNA delivery into the splenic lymphocytes, the splenic lymphocytes were infected by LV-sh4-1BB and LV-NC labeled with GFP. Successful lentiviral transfection was evidenced by green fluorescence under fluorescence microscopy 72 h after transduction (Figure 2). 

### 3.3. Lentivirus-Mediated Delivery of shRNA Inhibits 4-1BB Expression in the Splenic Lymphocytes *In Vitro*

**Figure 3:**
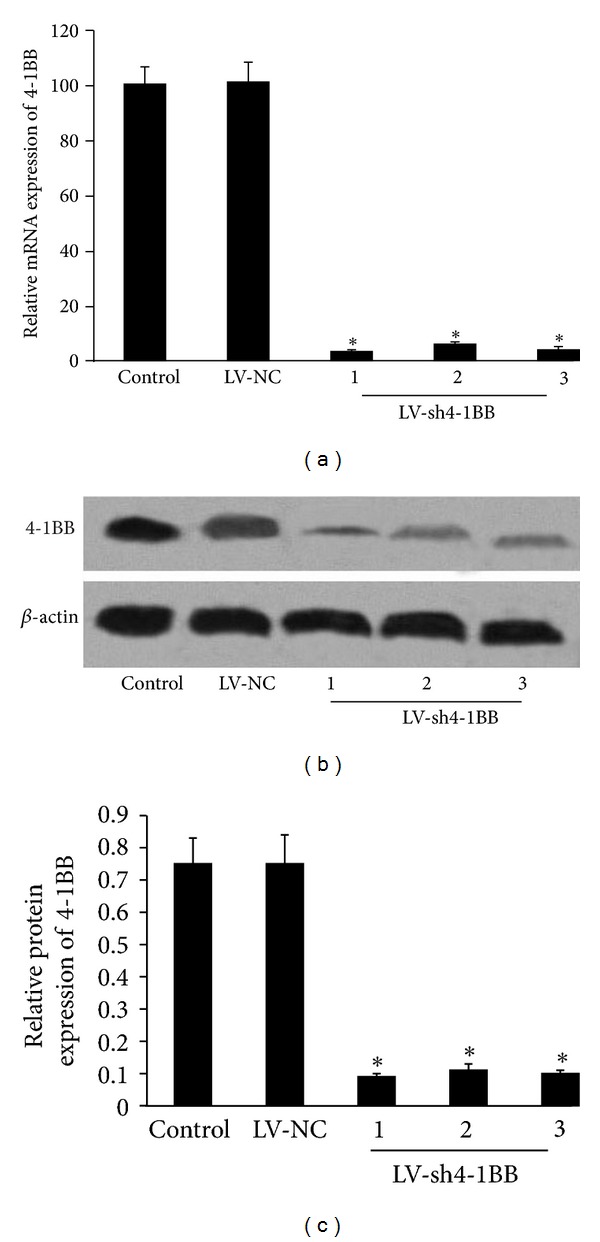
Effect of 4-1BB siRNA transduction on the expression of 4-1BB in the splenic lymphocytes. The recombination vector of lentivirus containing shRNA targeting the 4-1BB gene was constructed. Lymphocytes from BN rats were infected by recombinant LVs. ddH_2_O was used as the control. (a) Real-time PCR analysis of the mRNA level of 4-1BB gene in the splenic lymphocytes 72 h after transduction. (b and c) The protein level of 4-1BB gene in the splenic lymphocytes was showed by Western blot analysis 72 h after transduction. *β*-actin was used as an internal standard. LV-NC: negative-control shRNA group; LV-sh4-1BB1-3: pcDNA-CMV-sh4-1BB1-3-Lentivector group. Data were expressed as means ± SD. *P < 0.05 compared with the LV-NC group.

The mRNA expression of 4-1BB in the splenic lymphocytes was measured by real-time PCR 72 h after transduction. As shown in Figure 3(a), the mRNA expression of 4-1BB in the splenic lymphocytes was decreased approximately 96.8%, 94.1, and 95.2%, respectively, by LV-sh4-1BB1, LV-sh4-1BB2, and LV-sh4-1BB3 transduction compared to the empty LV-NC (all *P* < 0.05). These results indicated that the mRNA sequences corresponding to the 4-1BB gene shRNA were specific RNAi targets. 

Western blot analysis was performed 72 h after transduction. The results showed that LV-sh4-1BB1, LV-sh4-1BB2, and LV-sh4-1BB3 induced an 85.3%, 84.6, and 84.5% down-regulation of the 4-1BB protein level, respectively, compared with the empty LV-NC (all *P* < 0.05) (Figures 3(b) and 3(c)). So among three 4-1BB siRNAs, LV-sh4-1BB1 was selected as the best performing siRNA for use in the next study.

### 3.4. Lentivirus-Mediated Delivery of shRNA Targeting 4-1BB Gene Downregulated the 4-1BB Expression of the Splenic Lymphocytes Isolated from the Rats with Liver Transplantation

**Figure 4:**
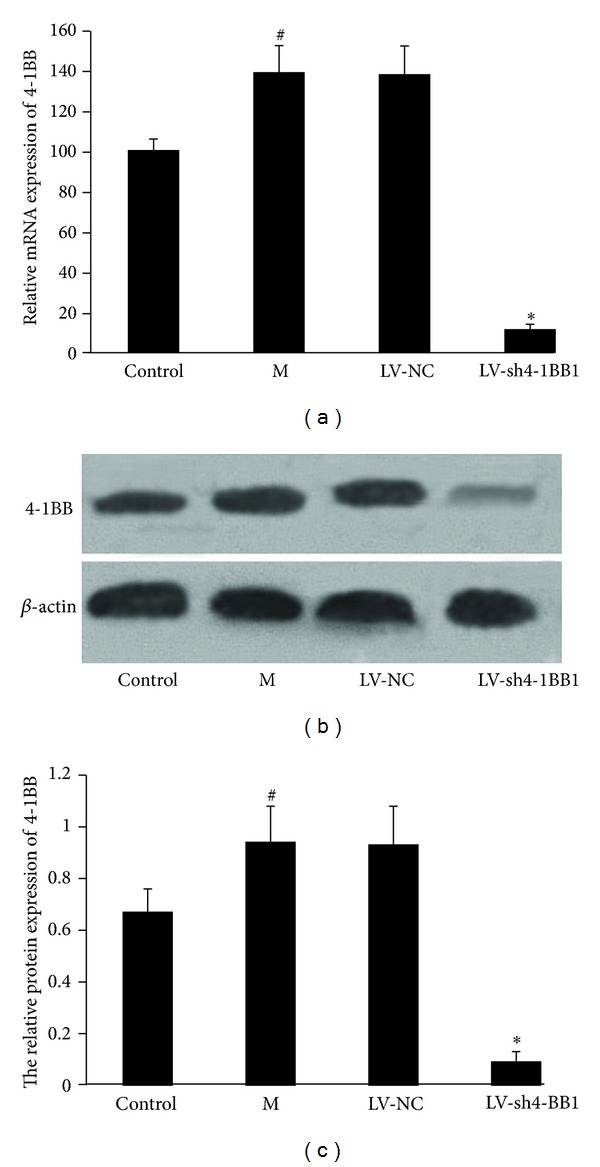
Effect of lentivirus-mediated delivery of shRNA targeting 4-1BB gene on 4-1BB expression of the splenic lymphocytes isolated from the recipient rats with liver transplantation. The recombination vector of lentivirus contains shRNA targeting the 4-1BB gene was constructed. The liver transplantation was performed using the two-cuff technique. BN recipient rats were infected by the recombinant LVs. The splenic lymphocytes from the recipient rats were gathered 7 days after transduction. Sham was used as the control. (a) Real-time PCR analysis of the mRNA level of 4-1BB gene in the splenic lymphocytes 7 days after transduction. (b and c) The protein level of 4-1BB gene in the splenic lymphocytes was showed by Western blot analysis 7 days after transduction. *β*-actin was used as an internal standard. M: the liver transplantation group; LV-NC: negative-control shRNA group; LV-sh4-1BB1: pcDNA-CMV-sh4-1BB1-Lentivector group. Data were expressed as means ± SD. ^#^ *P* < 0.05 compared with the sham (control) group; *P < 0.05 compared with the LV-NC group.

To evaluate the inhibition of 4-1BB mRNA expression in the splenic lymphocytes isolated from the recipient rats, real-time PCR was performed 7 days after transduction. As shown in Figure 4(a), the mRNA expression of 4-1BB in the model of liver transplantation group was significantly upregulated compared with the sham group (*P* < 0.05). LV-sh4-1BB1 transduction resulted in a 91.8% reduction of 4-1BB mRNA expression compared to the LV-NC groups (*P* < 0.05). Western blot analysis was performed 7 days after transduction. The results showed that the protein expression of 4-1BB was significantly upregulated in the model of liver transplantation group compared with the sham group (*P* < 0.05). LV-sh4-1BB1 transduction resulted in a 90.3% reduction of 4-1BB protein expression compared to the LV-NC groups (*P* < 0.05) (Figures 4(b) and 4(c)).

### 3.5. Lentivirus-Mediated Delivery of shRNA Targeting 4-1BB Gene Prolonged Recipient Survival in Rats with Liver Transplantation

**Figure 5:**
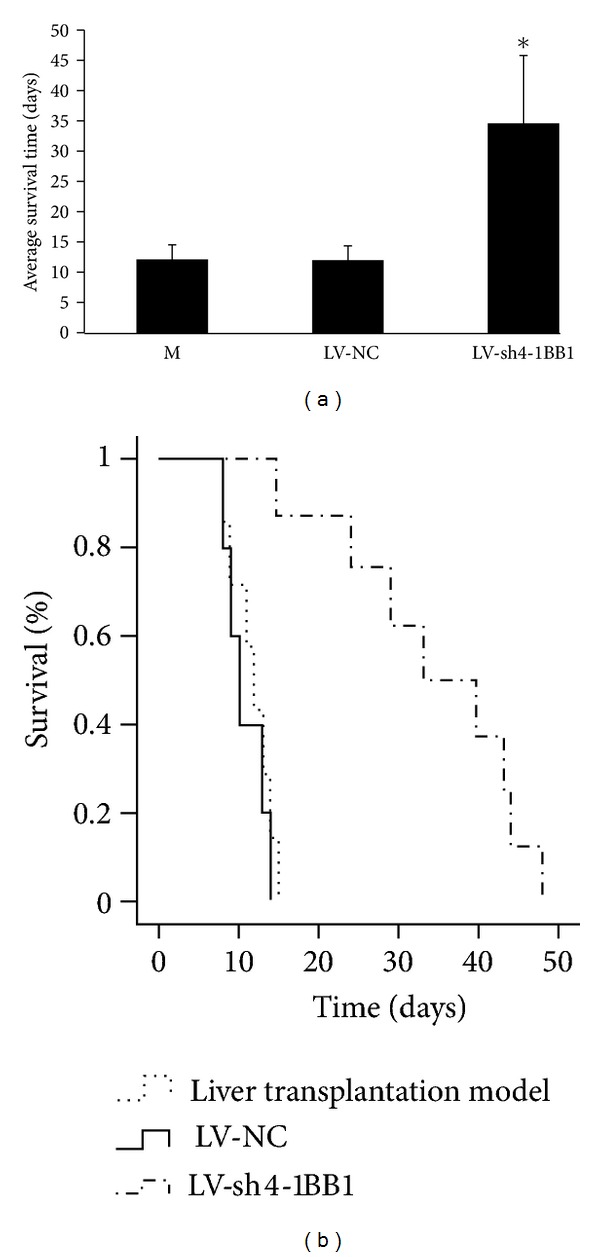
Effect of lentivirus-mediated delivery of shRNA targeting 4-1BB gene on the survival time in BN recipients rats after liver transplantation. The recombination vector of lentivirus contains shRNA targeting the 4-1BB gene (LV-sh4-1BB1) was constructed. The liver transplantation was performed using the two-cuff technique. BN recipient rats were infected by the recombinant LVs. The survival time of recipients rats was recorded and the percentage of survival was calculated. M: the liver transplantation model group; LV-NC: negative-control shRNA group; LV-sh4-1BB1: pcDNA-CMV-sh4-1BB1-Lentivector group. Data were expressed as means ± SD, *n* = 8. *P < 0.05 compared with the LV-NC group.

As shown in Figure 5, the survival time of BN recipients rats after liver transplantation was significantly increased in LV-sh4-1BB1 group compared with the LV-NC group (*P* < 0.05). The mean survival time (MST) of rats in LV-NC group was 12 days (range 8–14 days) and the MST of rats in LV-sh4-1BB1 group was 34.5 days (range 15–48 days). 12-day survival rate was 62.5% and 100.0% in LV-NC and LV-sh4-1BB1 group, respectively.

### 3.6. Lentivirus-Mediated Delivery of shRNA Targeting 4-1BB Gene Decreased the Level of Liver Function Damage in Recipient Rats with Liver Transplantation. 

As shown in Table 2, compared with the sham group, the plasma concentrations of AST, LDH, and ALT in liver transplantation model group were significantly increased 7 days after liver transplantation (*P* < 0.05). But the plasma concentrations of AST, LDH, and ALT in LV-sh4-1BB1 group were significantly decreased compared to the LV-NC group 7 days after liver transplantation (*P* < 0.05). 

### 3.7. Lentivirus-Mediated Delivery of shRNA Targeting 4-1BB Gene Decreased the Level of Plasma IL-2, IL-10, and IFN-*γ* in Rats with Liver Transplantation

The concentrations of plasma IL-2 and IFN-*γ* in liver transplantation model group were significantly increased 7 days after liver transplantation compared with the sham group. However, the plasma concentrations of plasma IL-2 and IFN-*γ* in LV-sh4-1BB1 group were significantly decreased compared with the LV-NC group 7 days after liver transplantation (*P* < 0.05) (Table 3).

### 3.8. Lentivirus-Mediated Delivery of shRNA Targeting 4-1BB Gene Alleviated the Injury of Liver Morphology in Recipient Rats with Liver Transplantation

**Figure 6:**
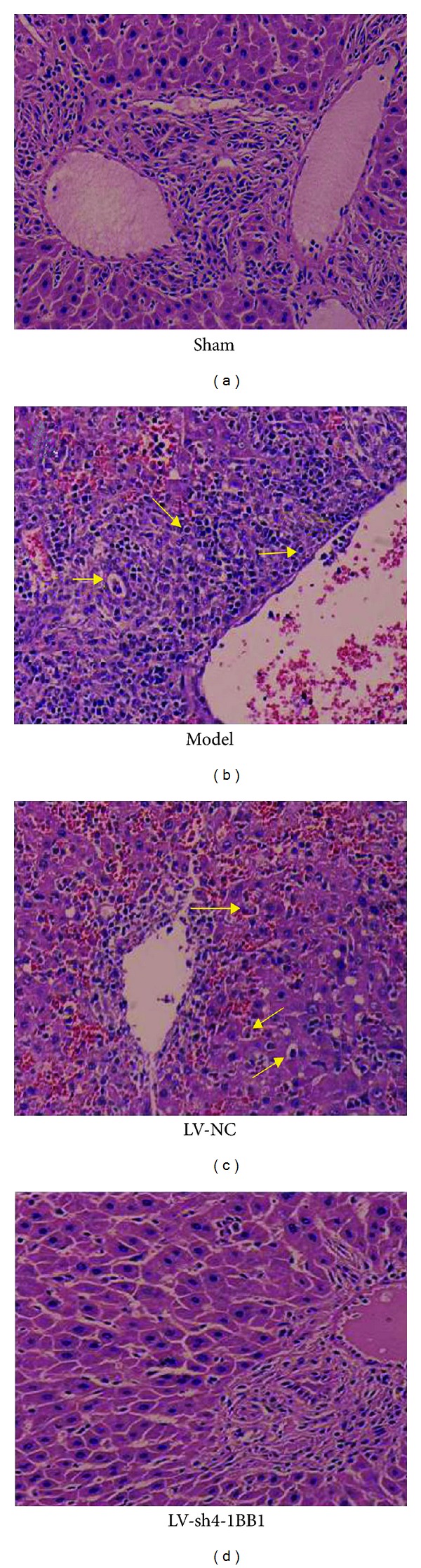
The pathology of liver in recipient rats with liver transplantation (HE, original magnification ×400). The recombination vector of lentivirus contains shRNA targeting the 4-1BB gene was constructed. The liver transplantation was performed using the two-cuff technique. BN recipient rats were infected by the recombinant LVs. The pathology of liver in recipient rats was observed by H-E staining 7 days after liver transplantation. Model: liver transplantation model group; LV-NC: negative-control shRNA group; LV-sh4-1BB1: pcDNA-CMV-sh4-1BB1-Lentivector group.

As shown in Figure 6, the severe portal lymphocyte infiltration, the injuries of the portal area and interlobular bile duct, cholangitis, and bridged necrosis in liver parenchyma were observed detected 7 days after liver transplantation in the liver transplantation model group. The rejection was significantly inhibited in the LV-sh4-1BB1 group compared with the LV-NC group. Mild-to-moderate portal inflammatory infiltration, lower grade of endothelialitis, mild bile duct injuries, and no evident hepatocyte necrosis were detected 7 days after liver transplantation in the LV-sh4-1BB1 group. 

**Figure 7:**
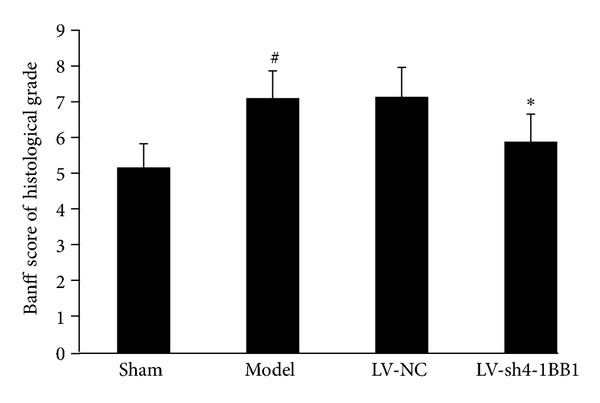
The Banff score of histological grade. Model: liver transplantation model group; LV-NC: negative-control shRNA group; LV-sh4-1BB1: pcDNA-CMV-sh4-1BB1-Lentivector group. Data were expressed as means ± SD, *n* = 8. ^#^ *P* < 0.05 compared with the sham group; *P < 0.05 compared with the LV-NC group.

The histological grade in Banff score in liver transplantation model group was significantly increased 7 days after liver transplantation compared with the sham control group (7.11 ± 0.78 versus 5.17 ± 0.68, *P* < 0.05) (Figure 7). Compared with the LV-NC group, the histological grade in Banff score in LV-sh4-1BB1 group was significantly decreased 7 days after liver transplantation (7.15 ± 0.84 versus 5.89 ± 0.79, *P* < 0.05).

**Figure 8:**
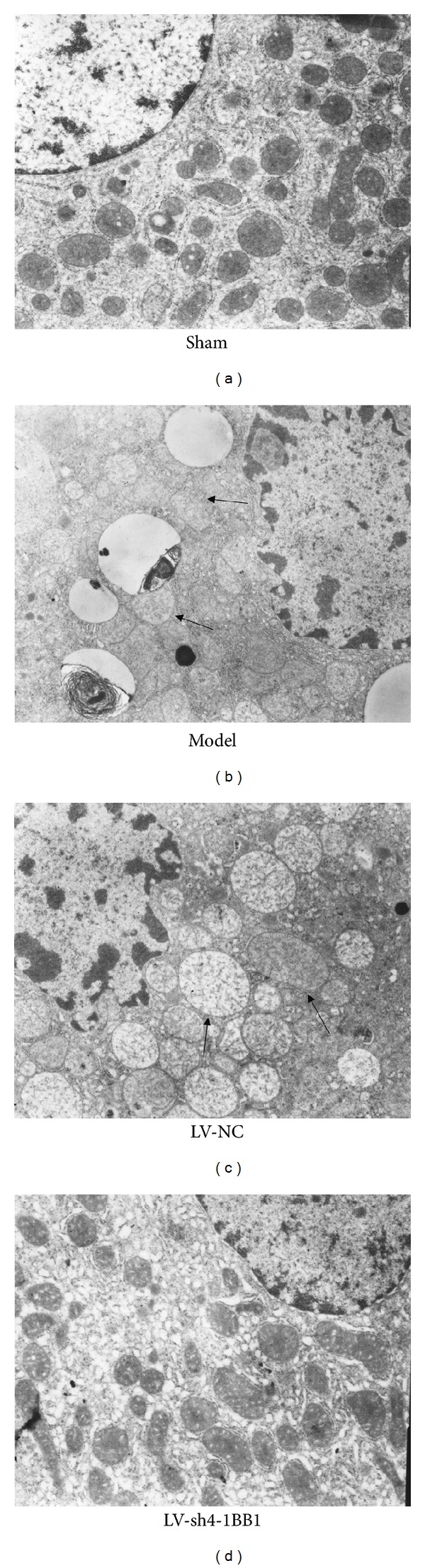
The electron micrographs of liver in recipient rats with liver transplantation (original magnification ×12000). The recombination vector of lentivirus contains shRNA targeting the 4-1BB gene was constructed. The liver transplantation was performed using the two-cuff technique. BN recipient rats were infected by the recombinant LVs. The ultrastructure of liver in recipient rats was observed by electron microscope 7 days after liver transplantation. Model: liver transplantation model group; LV-NC: negative-control shRNA group; LV-sh4-1BB1: pcDNA-CMV-sh4-1BB1-Lentivector group.

Electron micrographs showed that a typical early stage apoptosis of hepatocytes, swollen mitochondria, and dilatation of endoplasmic reticulum were observed in a majority of hepatocytes 7 days after liver transplantation in liver transplantation model group. But these phenomenons were not observed in LV-sh4-1BB1 group (Figure 8).

## 4. Discussion

The orthotopic liver transplantation has become the most effective therapy for the patients with end-stage liver disease. However, organ rejection is a thorny problem after transplantation, especially acute rejection which is the main cause of early dysfunction and retransplantation [21]. Therefore, it is very important to search new and effective methods to prevent and inhibit the organ rejection response in organ transplantation. 

Transplantation rejective response is principally mediated by T cells in the peripheral circulation and a variety of inflammatory stimuli that induce T cells to infiltrate the transplanted tissue [22]. CD4^+^ T cells secrete a number of cytokines which may induce cell infiltration in the graft after allogeneic recognition, and CD8^+^ T cells are involved in the direct cytotoxicity towards the liver graft [23]. Deletion, anergy, regulation/suppression, ignorance, or induction of activation induced T-cell death will be ways to suppress acute rejection and induce immune tolerance [24].

4-1BB is a family member of tumor necrosis factor receptor. 4-1BB is an important T-cell costimulatory molecule and expressed in activated cytolytic and helper T cells, as well as natural killers (NK) cells. The ligand of 4-1BB (4-1BBL) is expressed in B cells, macrophages, and dendritic cells. 4-1BB signaling preferentially promotes the proliferation and survival of CD8^+^ T cells and promotes the production of IL-2 in CD4^+^ T cells and prevents activation-induced cell death. 4-1BB is involved in the activation and survival of CD4^+^, CD8^+^, and NK cells [25]. The activation of T cells in the absence of costimulation is futile because T cells deprived of costimulatory signals enter a state of unresponsiveness or anergy.

The interaction of 4-1BB and 4-1BBL can activate an important costimulatory pathway which plays the diverse and important roles in immune response and organ transplantation tolerance. The previous studies showed that blocking the 4-1BB/4-1BBL signal pathway might modulate the secretion of Th1/Th2 cytokines and prolong the survival of the grafts [26]. It was reported that administration of agonistic anti-4-1BB monoclonal antibody (mAB) prevented the development of various autoimmune and nonautoimmune conditions *in vivo* [27, 28]. Our previous results also suggested the blockade of the 4-1BB/4-1BBL costimulatory pathway with 4-1BBL monoclonal antibody attenuated the acute rejection in recipient rats with the liver transplantation [11].

RNAi-based gene silencing is more rapid and cost-effective compared with the gene knockout techniques. RNA interference (RNAi) is a powerful tool to induce loss-of-function phenotypes by posttranscriptional silencing of gene expression. Viral delivery of short hairpin RNA (shRNA) expression cassettes allows efficient transduction in tissues such as immunological cell *in vivo* [29]. The knockdown of gene expression has been achieved using lentiviral vector constructs that express shRNAs within vector-infected cells. The lentiviral vector system provided useful tools for elucidating gene function by analysis of loss-of-function phenotypes and for exploring the application of RNAi in gene therapy [30]. In our study, The recombination vector of lentivirus containing shRNA targeting the 4-1BB gene was successfully constructed. The liver transplantation was performed using the two-cuff technique. BN recipient rats were infected by the recombinant LVs. The results showed that gene silencing of 4-1BB by RNA interference downregulated the 4-1BB gene expression of the splenic lymphocytes isolated from the recipient rats with liver transplantation. Our results also showed that lentivirus-mediated delivery of shRNA targeting 4-1BB gene prolonged the survival time of recipient rats. These results suggested that the silencing of 4-1BB gene by RNA interference was successful in the recipient rats and useful for the liver transplantation. 

The acute transplantation rejective response is the important cause of retransplantation and is principally mediated by T cells [31]. Cytokines including IL-2, IL-10, and INF-*γ* can accelerate the T-cell mediated immune response, while IL-4, 5, and 6 can be helpful for B-cell mediated humoral immunity. Increase of cytokines is the marker of the acute transplantation rejective response [32, 33]. Our results showed that gene silencing of 4-1BB by RNA interference decreased the levels of plasma IL-2 and INF-*γ* seven days after liver transplantation. These illuminated that gene silencing of 4-1BB inhibited the T cell-mediated acute rejection in recipient rats with liver transplantation. 

Histopathology is the gold standard for diagnosing graft rejection after transplantation [34]. There are the infiltration of inflammatory cells to portal area including activated lymphocytes, neutrophils and acidophils, inflammation of endotheliocytes under the portal vein and central vein, and the inflammation and injury of the bile duct in the acute rejection of liver transplantation [35, 36]. The dysfunction of the liver should be observed in the acute rejection of liver transplantation. In our study, the results showed that gene silencing of 4-1BB by RNA interference decreased the plasma levels of liver injury markers including AST, ALT, and BIL and alleviated the injury of liver morphology in recipient rats with liver transplantation. Our experiment strongly suggested gene silencing of 4-1BB by RNA interference prevented the liver injury induced by the acute rejection in rats with liver transplantation.

In conclusion, we have demonstrated that gene silencing of 4-1BB by RNA interference inhibits the acute rejection in rats with liver transplantation and it is a promising strategy to prevent progression of graft rejection.

## Figures and Tables

**Table 1:** Sense and antisense oligomers of shRNA targeting the 4-1BB gene of rats.

shRNA symbol	Target sequence	Start position	shRNA strand
shRNA1	GCTGTTACAAC ATGGTGGTCA	11	sense 5′-CACCGCTGTTACAACATGGTGGTCATTCAAGAGATGA CCACCATGTTGTAACAGCTTTTTTG-3′ antisense 5′-ACCTTGATTCCAACGGTAACCATCATCTCTTGAAGGA TACCTTCAAGCATTCCGGAGGATTA-3′

shRNA2	GCAGTAAATAC CCTCCGGTCT	110	sense 5′-CACCGCAGTAAATACCCTCCGGTCTTTCAAGAGAAG ACCGGAGGGTATTTACTGCTTTTTTG -3′ antisense 5′-AACCCCTAGGGCTTAAGCAATCCAGTCTCTTGAACCA TTTGGCACTTAAGCCATTGACTGAC-3′

shRNA3	GCAGAGTGTGT CAAGGCTATT	191	sense 5′-CACCGCAGAGTGTGTCAAGGCTATTTCAAGAGAAT AGCCTTCGAGCACACTCTGCTTTTTTG -3′ antisense 5′-AGGTTACCGGGATCCTTGAACAATACTCTTGAACA ACCCGGTTTACCAAATACCGGAATTGG-3′

shRNANC	ATTGGACCAAG TGGTTCATAGC		sense 5′-CCTGACGATTAAGGACTAGGTCAGCTTCAAGAGACCC CTTAAAGCTTTCGGACGTCGCGAAC-3′ antisense 5′-AAGTACCAACTACATCTGGGAACTTCTCTTGAAACCC TGAATGGGTCATCGTAACTACAAC-3′

**Table 2:** The levels of AST, LDH, ALT, and T-BIL in plasma of BN recipients rats 7 days after liver transplantation.

Groups	ALT (U/L)	AST (U/L)	LDH (U/L)	T-BIL (mg/L)
Sham (Control)	292.43 ± 32.41	428.39 ± 34.66	859.34 ± 94.36	75.39 ± 8.12
Model	580.52 ± 51.96^#^	738.11 ± 75.63^#^	1404.65 ± 168.74^#^	83.94 ± 9.53
LV-NC	577.21 ± 61.14	784.7 ± 47.85	1553.19 ± 125.83	84.38 ± 10.46
LV-sh4-1BB1	333.26 ± 39.92*	457.7 ± 43.61*	942.81 ± 83.17*	76.84 ± 8.43

The recombination vector of lentivirus containing shRNA targeting the 4-1BB gene was constructed. The liver transplantation was performed using the two-cuff technique. BN recipient rats were infected by the recombinant LVs. The blood samples were gathered 7 days after liver transplantation. The liver function markers were tested by using the automatic biochemical meter. Model: liver transplantation model group; LV-NC: negative-control shRNA group; LV-sh4-1BB1: pcDNA-CMV-sh4-1BB-Lentivector1 group. Data were expressed as means ± SD, *n* = 8. ^#^ *P* < 0.05 compared with the sham (control) group; *P < 0.05 compared with the LV-NC group.

**Table 3:** The levels of IL-2, IL-10, and IFN-*γ* in plasma of BN recipients rats 7 days after liver transplantation.

Groups	IL-2 (pg/mL)	IL-10 (pg/mL)	IFN-*γ* (pg/mL)
Sham (Control)	71.32 ± 8.67	16.85 ± 2.03	52.49 ± 6.75
Model	139.15 ± 13.73^#^	15.47 ± 1.27	86.2 ± 8.27^#^
LV-NC	142.86 ± 16.58	18.35 ± 2.06	80.3 ± 11.64
LV-sh4-1BB1	78.05 ± 8.64*	16.59 ± 1.85	57.86 ± 8.33*

The recombination vector of lentivirus contains shRNA targeting the 4-1BB gene was constructed. The liver transplantation was performed using the two-cuff technique. BN recipient rats were infected by the recombinant LVs. The blood samples were gathered 7 days after liver transplantation. The concentrations of interleukin-2 (IL-2), IL-10, and interferon-*γ* (IFN-*γ*) in plasma were tested using enzyme-linked immunosorbent assay (ELISA). Model: liver transplantation model group; LV-NC: negative-control shRNA group; LV-sh4-1BB1: pcDNA-CMV-sh4-1BB-Lentivector1 group. Data were expressed as means ± SD, *n* = 8. ^#^ *P* < 0.05 compared with the sham (control) group; *P < 0.05 compared with the LV-NC group.

## References

[1] Goronzy JJ, Weyand CM (2008). T-cell co-stimulatory pathways in autoimmunity. *Arthritis Research and Therapy*.

[2] Kornete M, Piccirillo CA (2011). Critical co-stimulatory pathways in the stability of Foxp3^+^ Treg cell homeostasis in type I diabetes. *Autoimmunity Reviews*.

[3] Palazón A, Martínez-Forero I, Teijeira A (2012). The HIF-1*α* hypoxia response in tumor-infiltrating T lymphocytes induces functional CD137 (4-1BB) for immunotherapy. *Cancer Discovery*.

[4] Teschner D, Wenzel G, Distler E (2011). In vitro stimulation and expansion of human tumour-reactive CD8^+^ cytotoxic t lymphocytes by anti-CD3/CD28/CD137 magnetic beads. *Scandinavian Journal of Immunology*.

[5] Hernandez-Chacon JA, Li Y, Wu RC (2011). Costimulation through the CD137/4-1BB pathway protects human melanoma tumor-infiltrating lymphocytes from activation-induced cell death and enhances antitumor effector function. *Journal of Immunotherapy*.

[6] Chen HW, Liao CH, Ying C, Chang CJ, Lin CM (2006). Ex vivo expansion of dendritic-cell-activated antigen-specific CD4^+^ T cells with anti-CD3/CD28, interleukin-7, and interleukin-15: potential for adoptive T cell immunotherapy. *Clinical Immunology*.

[7] Stefanidis C, Callebaut G, Ngatchou W (2012). The role of biventricular assistance in primary graft failure after heart transplantation. *Hellenic Journal of Cardiology*.

[8] Glanemann M, Gaebelein G, Nussler N (2009). Transplantation of monocyte-derived hepatocyte-like cells (NeoHeps) improves survival in a model of acute liver failure. *Annals of Surgery*.

[9] Asai T, Choi BK, Kwon PM (2007). Blockade of the 4-1BB (CD137)/4-1BBL and/or CD28/CD80/CD86 costimulatory pathways promotes corneal allograft survival in mice. *Immunology*.

[10] Huang BJ, Yin H, Huang YF (2006). Gene therapy using adenoviral vector encoding 4-1BBIg gene significantly prolonged murine cardiac allograft survival. *Transplant Immunology*.

[11] Qin L, Guan HG, Zhou XJ, Yin J, Lan J, Qian HX (2010). Blockade of 4-1BB/4-1BB ligand interactions prevents acute rejection in rat liver transplantation. *Chinese Medical Journal*.

[12] Wu N, Yu AB, Zhu HB, Lin XK (2012). Effective silencing of Sry gene with RNA interference in developing mouse embryos resulted in feminization of XY gonad. *Journal of Biomedicine and Biotechnology*.

[13] Yang T, Zhang B, Pat BK, Wei MQ, Gobe GC (2010). Lentiviral-mediated RNA interference against TGF-beta receptor type II in renal epithelial and fibroblast cell populations in vitro demonstrates regulated renal fibrogenesis that is more efficient than a nonlentiviral vector. *Journal of Biomedicine and Biotechnology*.

[14] Ui-Tei K, Naito Y, Takahashi F (2004). Guidelines for the selection of highly effective siRNA sequences for mammalian and chick RNA interference. *Nucleic Acids Research*.

[15] Kuo YC, Weng SC, Chou CJ, Chang TT, Tsai WJ (2003). Activation and proliferation signals in primary human T lymphocytes inhibited by ergosterol peroxide isolated from Cordyceps cicadae. *British Journal of Pharmacology*.

[16] Muscarella LA, Guarnieri V, Coco M (2010). Small deletion at the 7q21.2 locus in a CCM family detected by real-time quantitative PCR. *Journal of Biomedicine and Biotechnology*.

[17] Kim CS, Kim JG, Lee BJ (2011). Deficiency for costimulatory receptor 4-1BB protects against obesity-induced inflammation and metabolic disorders. *Diabetes*.

[18] Hori T, Gardner LB, Chen F (2012). Impact of hepatic arterial reconstruction on orthotopic liver transplantation in the rat. *Journal of Investigative Surgery*.

[19] Demetris AJ, Batts KP, Dhillon AP (1997). Banff schema for grading liver allograft rejection: an international consensus document. *Hepatology*.

[20] Sanei MH, Schiano TD, Sempoux C, Fan C, Fiel MI (2011). Acute cellular rejection resulting in sinusoidal obstruction syndrome and ascites postliver transplantation. *Transplantation*.

[21] Fosby B, Karlsen TH, Melum E (2012). Recurrence and rejection in liver transplantation for primary sclerosing cholangitis. *World Journal of Gastroenterology*.

[22] Gras S, Kjer-Nielsen L, Chen Z, Rossjohn J, McCluskey J (2011). The structural bases of direct T-cell allorecognition: implications for T-cell-mediated transplant rejection. *Immunology and Cell Biology*.

[23] Pérez-Flores I, Sánchez-Fructuoso A, Santiago JL (2009). Intracellular ATP levels in CD4^+^ lymphocytes are a risk marker of rejection and infection in renal graft recipients. *Transplantation Proceedings*.

[24] McKallip RJ, Do Y, Fisher MT, Robertson JL, Nagarkatti PS, Nagarkatti M (2002). Role of CD44 in activation-induced cell death: CD44-deficient mice exhibit enhanced T cell response to conventional and superantigens. *International Immunology*.

[25] Choi BK, Bae JS, Choi EM (2004). 4-1BB-dependent inhibition of immunosuppression by activated CD4^+^, CD25^+^ T cells. *Journal of Leukocyte Biology*.

[26] Xu K, Li C, Pan X, Du B (2007). Study of relieving graft-versus-host disease by blocking CD137-CD137 ligand costimulatory pathway in vitro. *International Journal of Hematology*.

[27] Kim J, Choi WS, La S (2005). Stimulation with 4-1BB (CD137) inhibits chronic graft-versus-host disease by inducing activation-induced cell death of donor CD4^+^ T cells. *Blood*.

[28] Ganguly S, Liu J, Pillai VB, Mittler RS, Amara RR (2010). Adjuvantive effects of anti-4-1BB agonist Ab and 4-1BBL DNA for a HIV-1 Gag DNA vaccine: different effects on cellular and humoral immunity. *Vaccine*.

[29] Meliopoulos VA, Andersen LE, Birrer KF (2012). Host gene targets for novel influenza therapies elucidated by high-throughput RNA interference screens. *The FASEB Journal*.

[30] Hutson TH, Foster E, Dawes JM, Hindges R, Yáñez-Muñoz RJ, Moon LD (2012). Lentiviral vectors encoding short hairpin RNAs efficiently transduce and knockdown LINGO-1 but induce an interferon response and cytotoxicity in central nervous system neurones. *Journal of Gene Medicine*.

[31] del Rio ML, Kurtz J, Perez-Martinez C, Ghosh A, Perez-Simon JA, Rodriguez-Barbosa JI (2011). B- and T-lymphocyte attenuator targeting protects against the acute phase of graft versus host reaction by inhibiting donor anti-host cytotoxicity. *Transplantation*.

[32] Chen G, Mi J, Xiao MZ, Fu YR (2012). PDIA3 mRNA expression and IL-2, IL-4, IL-6, and CRP levels of acute kidney allograft rejection in rat. *Molecular Biology Reports*.

[33] Karimi MH, Daneshmandi S, Pourfathollah AA (2011). Association of IL-6 promoter and IFN-*γ* gene polymorphisms with acute rejection of liver transplantation. *Molecular Biology Reports*.

[34] Kulkarni P, Uppin MS, Prayaga AK, Das U, Murthy KVD (2011). Renal allograft pathology with C4d immunostaining in patients with graft dysfunction. *Indian Journal of Nephrology*.

[35] Sanada Y, Mizuta K, Urahashi T (2012). Co-occurrence of nonanastomotic biliary stricture and acute cellular rejection in liver transplant. *Experimental and Clinical Transplantation*.

[36] Wyatt JI (2010). Liver transplant pathology—messages for the non-specialist. *Histopathology*.

